# Multiple pseudoaneurysms in complicated infective endocarditis: importance of the Yin–Yang sign and multimodality imaging—case report

**DOI:** 10.1093/ehjcr/ytaf589

**Published:** 2025-11-17

**Authors:** César Augusto Rojas-Sánchez, Gabriela Meléndez-Ramírez, Zaee Alvarado-Toledo, Gladis Faustino-Maravilla, Heberto Aquino-Bruno

**Affiliations:** Department of Cardiology, Centro Médico Nacional 20 de Noviembre, Av. Felix Cuevas #540, Col. Del Valle, Del. Benito Juarez, Mexico City 03100, Mexico; Department of Cardiovascular Imaging, Centro Médico Nacional 20 de Noviembre, Av. Felix Cuevas #540, Col. Del Valle, Del. Benito Juarez, Mexico City 03100, Mexico; Department of Pediatric Cardiology, Centro Médico Nacional 20 de Noviembre, Av. Felix Cuevas #540, Col. Del Valle, Del. Benito Juarez, Mexico City 03100, Mexico; Department of Echocardiography, Centro Médico Nacional 20 de Noviembre, Av. Felix Cuevas #540, Col. Del Valle, Del. Benito Juarez, Mexico City 03100, Mexico; Department of Interventional Cardiology, Centro Médico Nacional 20 de Noviembre, Av. Felix Cuevas #540, Col. Del Valle, Del. Benito Juarez, Mexico City 03100, Mexico

**Keywords:** Infective endocarditis, Pseudoaneurysm, Yin–Yang sign, Case report

## Abstract

**Background:**

Infective endocarditis (IE) continues to show a rising global incidence and significant associated mortality. Among its complications, pseudoaneurysms (pAs) are peri-annular lesions resulting from localized tissue destruction caused by infection, leading to a focal dilatation. These lesions usually occur as isolated findings, and the presence of multiple pAs is rare. Importantly, pAs are recognized predictors of in-hospital mortality.

**Case summary:**

We describe three distinct patients exhibiting IE complicated with multiple pAs with particular and unique features. Patient 1 is a 49-year-old woman with pre-existing bicuspid aortic valve (BAV), with fever and dyspnoea; two pAs were identified (in the aortic root and mitral–aortic intervalvular fibrosa) and were successfully treated with surgical resection. Patient 2 is a 51-year-old man with past history of a Bentall procedure, presented with 3-month history of fever. Two large pAs were found in the aortic root; despite successful surgical repair, the patient died secondary of refractory vasoplegic shock. Patient 3 is a 3-year-old boy, with a known BAV with 4 weeks of high-degree fever. Three pAs were identified (two in the aortic root and one in the mitral valve) along with two other congenital heart defects. Unfortunately, despite full medical treatment, patient died due to refractory cardiogenic shock.

**Discussion:**

Multimodality imaging served as a cornerstone for the accurate diagnosis, anatomical localization, and extension of pAs in all three patients. Echocardiography helped to reveal the echo-free spaces with systolic expansion and diastolic collapse, a feature that was also observed in the cardiac angio-computed tomography. Notably, the presence of the Yin–Yang sign on colour Doppler imaging served as a key diagnostic clue and should be carefully sought when evaluating patients with IE.

Learning points

- Pseudoaneurysms may present as multiple lesions in a single case; recognizing an echo-free pulsatile space with flow within it is crucial in the context of infective endocarditis.
- The Yin–Yang sign is a valuable diagnostic clue, characterized by bidirectional flow on colour Doppler.

## Introduction

Infective endocarditis (IE) still has a great burden, with increasing incidence and a worldwide mortality rate of 30%.^1^ Independent predictors of in-hospital death are septic shock and peri-annular complications^2^; including valvular destruction or perforation, annular abscess, and pseudoaneurysm (pA).

Pseudoaneurysms are a quite rare complication of IE, defined as a contained rupture of the peri-annular tissue generating a focal dilatation resulting from localized destruction, without involvement of all three layers of the vessel or annular wall. Unlike vascular pAs, those occurring in IE can also develop in peri-annular regions such as the mitral–aortic intervalvular fibrosa (MAIV-F), where no true arterial wall is present. These lesions are most often encountered as solitary findings. This case series documents the occurrence of multiple pAs across three distinct patients, highlighting their unique features.

## Summary figure

**Figure:**
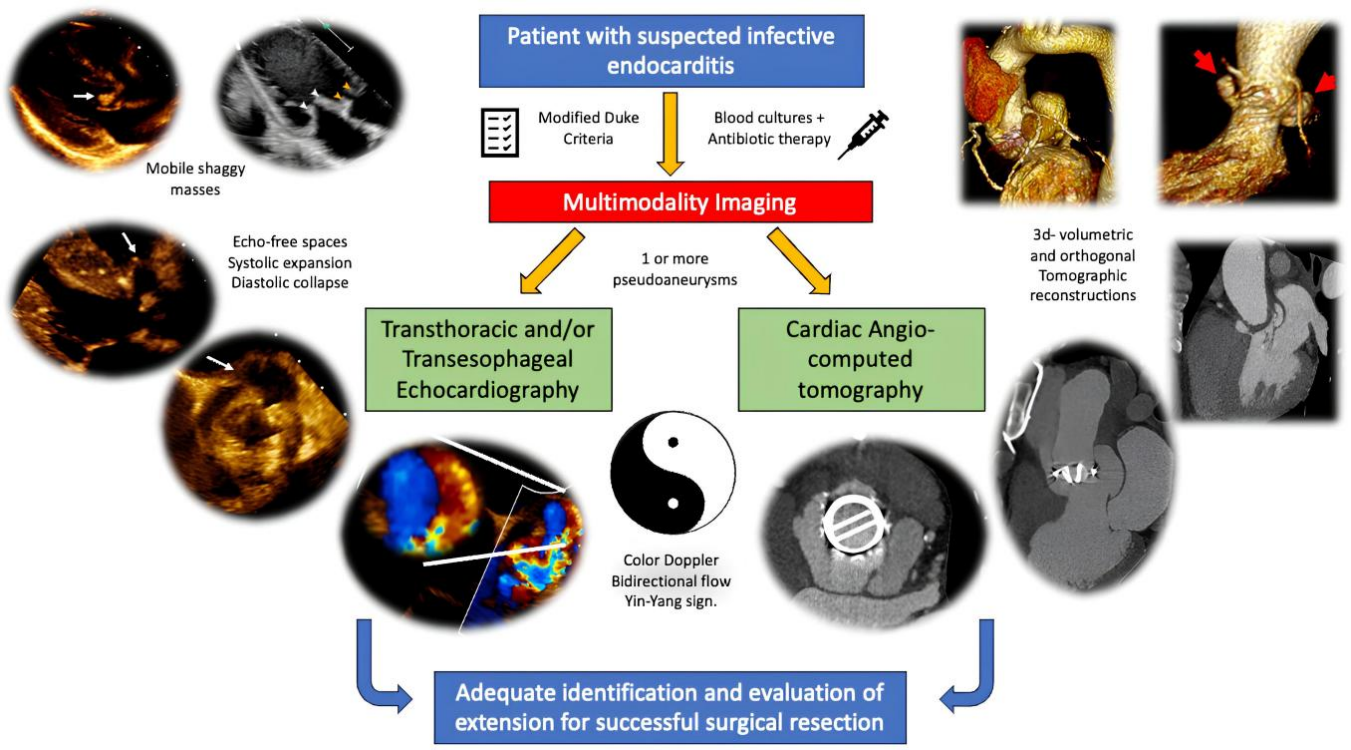


## Patient 1

**Figure 1:**
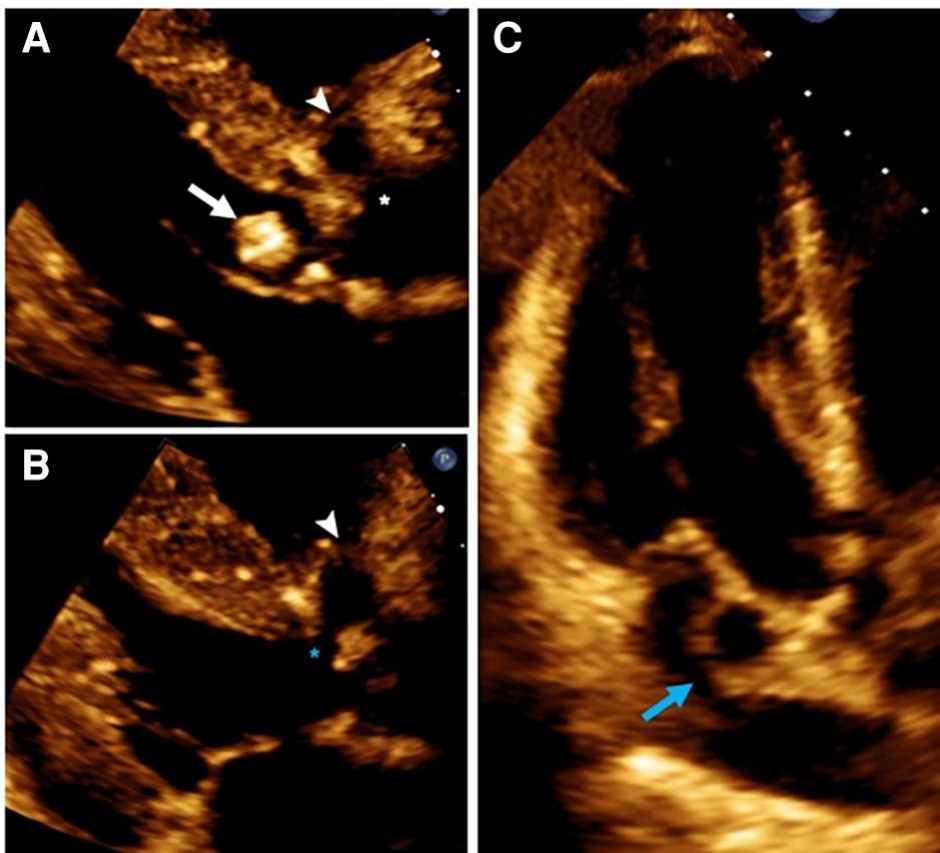
Transthoracic echocardiography. (*A*) Parasternal Long Axis View (PLAX) during diastole showing a mobile vegetation on aortic valve (*white arrow*) and anterior pseudoaneurysm (*white arrow head*), with aortic fistulization (*white asterisk*). (*B*) PLAX showing during systole anterior pseudoaneurysm and fistulization to left ventricular outflow tract (*blue asterisk*). (*C*) Three-chamber view during systole demonstrating pseudoaneurysm of the mitral–aortic intervalvular fibrosa (*blue arrow*).

**Figure 2:**
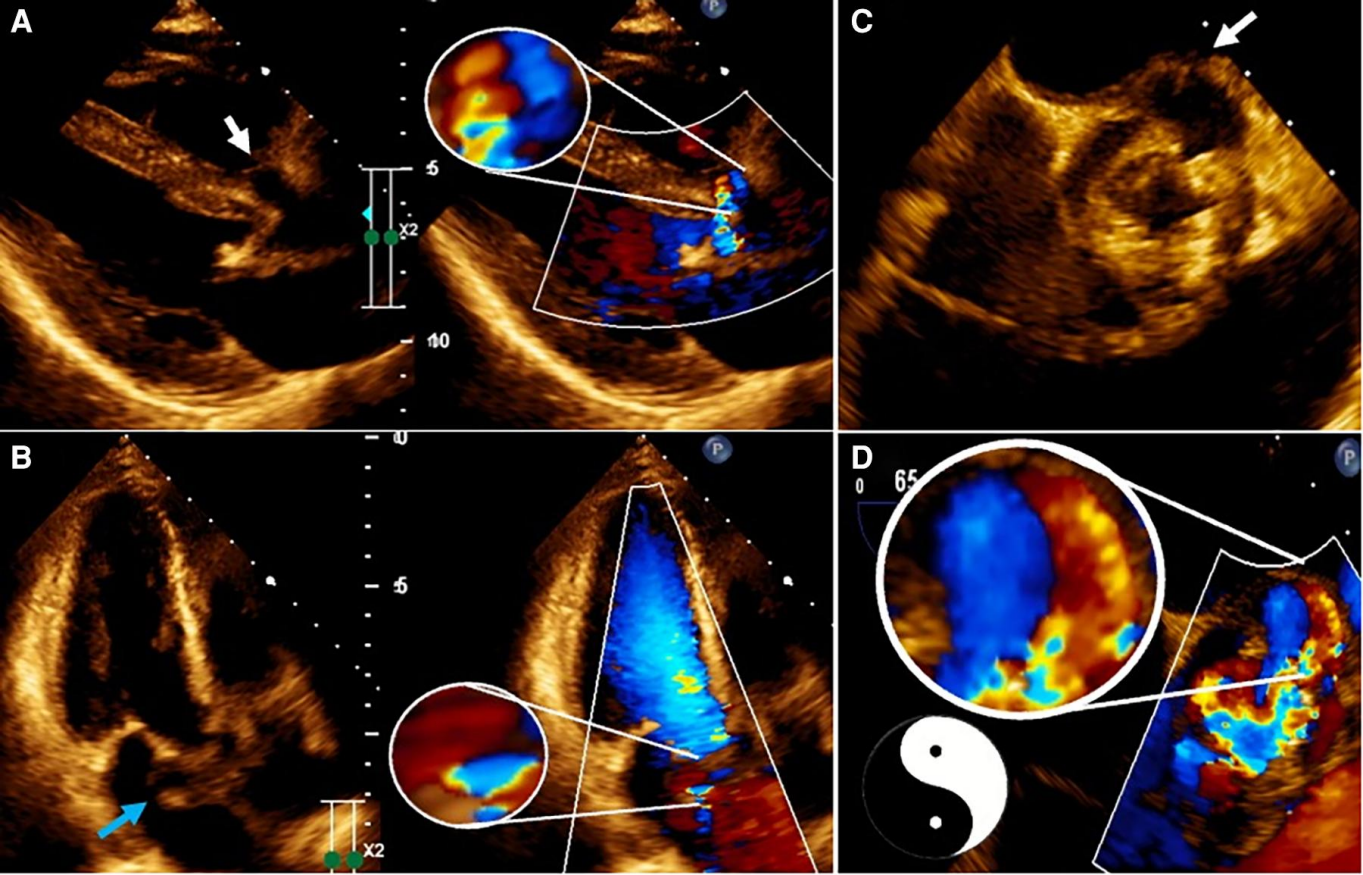
Transthoracic echocardiography (*A–B*), and transoesophageal echocardiography (*C*: 45° view; *D*:65° view) with dual-image colour Doppler illustrating bidirectional flow within aortic annulus pseudoaneurysm (*white arrows*) and mitral–aortic intervalvular fibrosa pseudoaneurysm (*blue arrow*).

**Figure 3:**
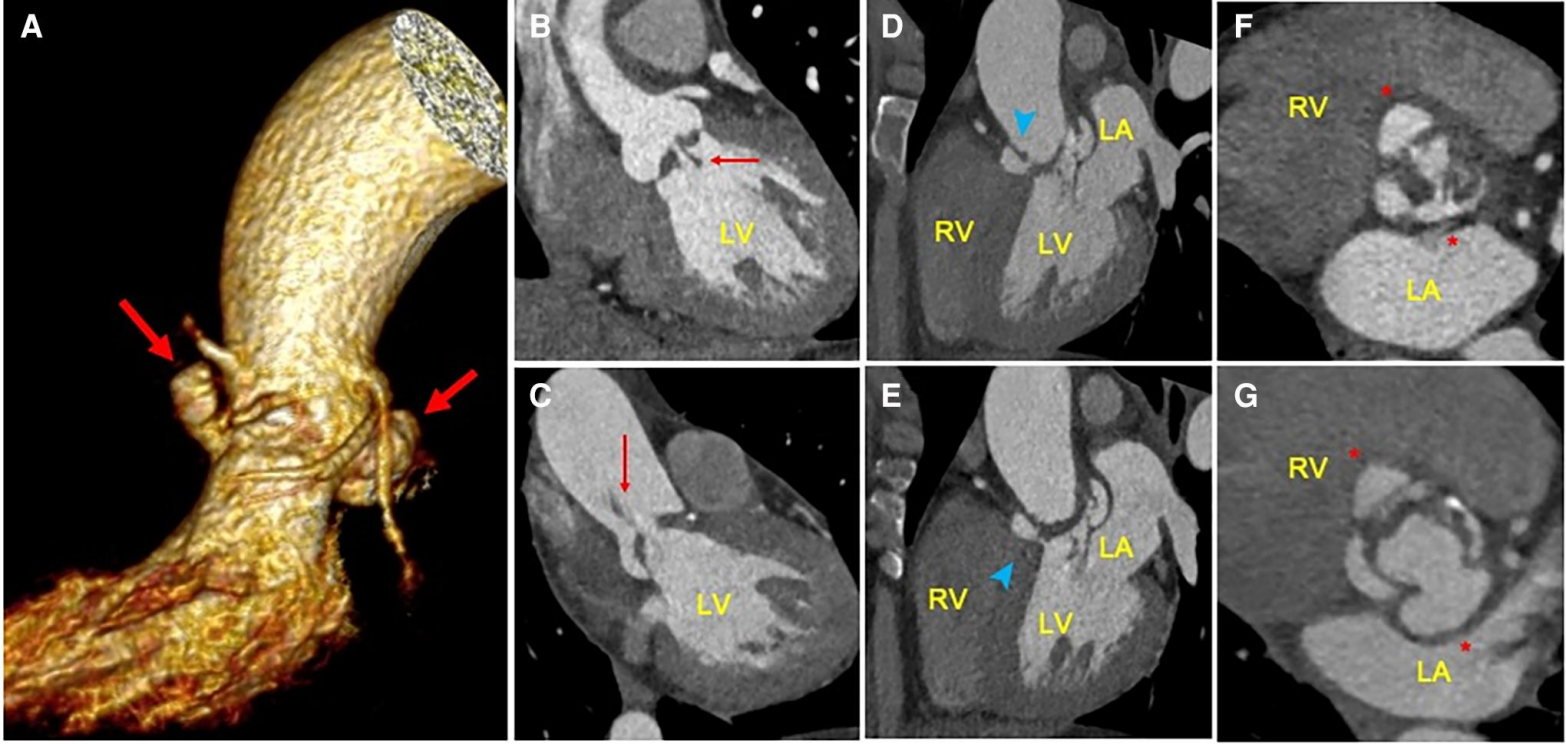
(*A*) Three-dimensional volumetric tomographic reconstruction showing left ventricular outflow tract and mitral–aortic intervalvular fibrosa pseudoaneurysm (*red thick arrows*). (*B–G*) Orthogonal reconstructions demonstrating two mobile vegetations (*red thin arrows*) *(B–C). (D–E)* Double fistulization (*blue arrow heads*) of pseudoaneurysm to the aortic root (*D*) and left ventricular outflow tract (*E*). (*F–G*) Partial diastolic collapse (*F*), and systolic expansion (*G*) of both pseudoaneurysms (*red asterisks*)*. LV*, left ventricle; *RV*, right ventricle; *LA*, left atrium.

A 49-year-old woman with a past history of a non-specific heart murmur without diagnostic evaluation at the age of 19 presented to the emergency department with 1 month of fever and dyspnoea. A transthoracic echocardiography (TTE) revealed stenosis and regurgitation of a bicuspid aortic valve (BAV), a mobile shaggy mass on the non-coronary cusp (*Figure 1A*), and pulsatile echo-free spaces in the aortic root and the MAIV-F (*Figure 1A–C*, Supplementary material online, *Videos S1*–S2). Transoesophageal echocardiography (TEE) identified a pA in the right sinus of Valsalva and the MAIV-F (see Supplementary material online, *Video S3*). Colour Doppler demonstrated bidirectional flow (Yin–Yang sign) (*Figure 2*, Supplementary material online, *Videos S1*, *S4*). Subsequently, a cardiac angio-computed tomography (angio-CT) revealed two mobile vegetations (*Figure 3B* and *C*) and confirmed two pAs: one in the right sinus of Valsalva measuring 11 × 7 mm with double fistulization to the left ventricular outflow tract (LVOT) and aortic root and another measuring 16 × 11 mm in the MAIV-F (*Figure 3A, D–G*). Empiric antibiotic therapy was initiated and later adjusted to targeted therapy against *Streptococcus infantarius*. The patient underwent urgent surgical resection of the vegetations and pAs, followed by reconstruction of the aortic root and LVOT with autologous pericardium and mechanical aortic valve replacement. Six months later, the patient remained asymptomatic and with normal prosthetic valve function.

## Patient 2

A 51-year-old man with a past history of ascending aortic aneurysm treated with a mechanical aortic valve replacement (St. Jude 31 mm) and Bentall procedure 10 years earlier, with no further follow up, presented to the emergency department with a 3-month history of recurrent fever. Potential sources of infection were excluded. Intravenous antibiotics were initiated after blood cultures were obtained. A TTE and TEE could not be performed due to poor transthoracic windows and the patient’s refusal to provide consent, respectively.

**Figure 4:**
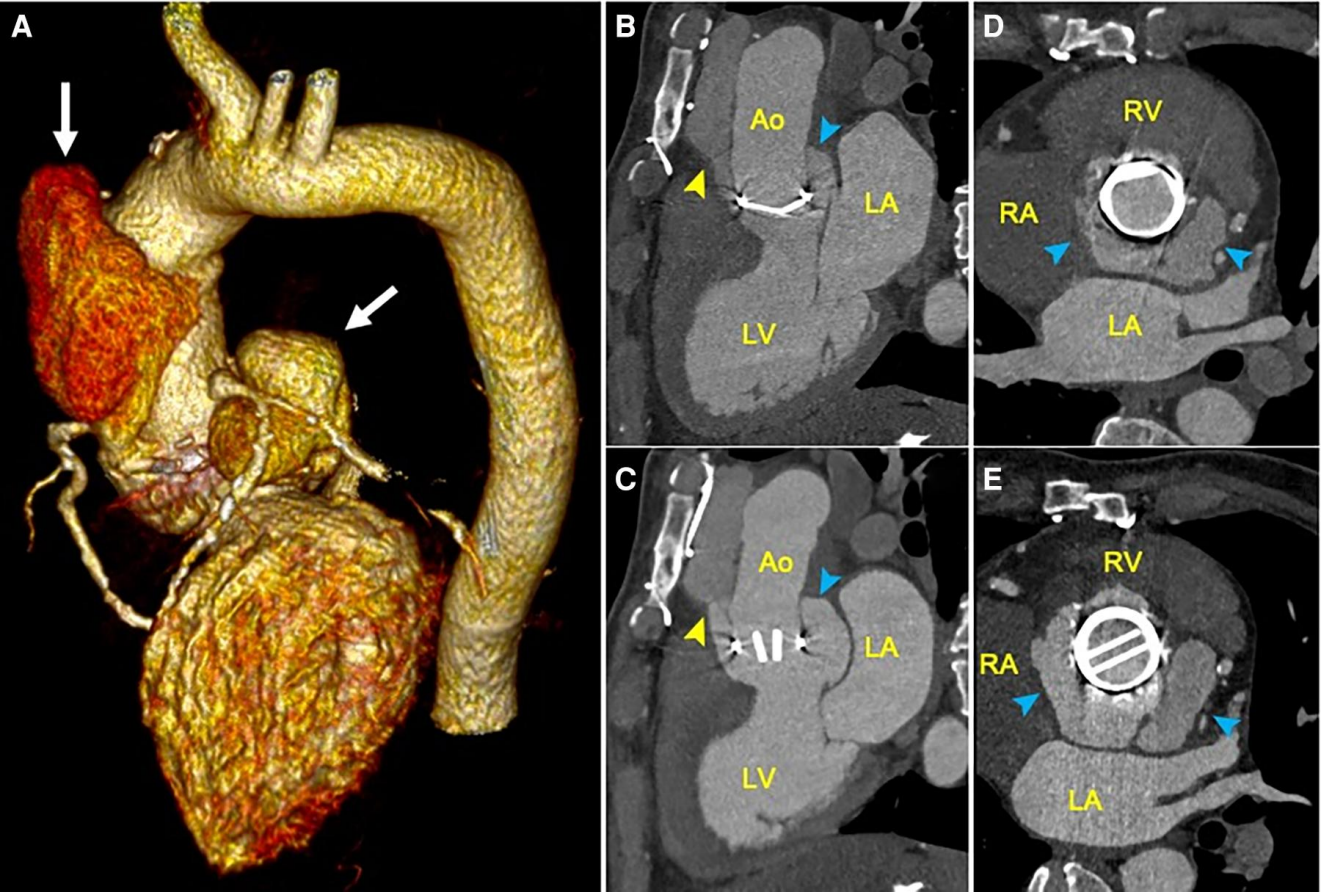
(*A*) Three-dimensional volumetric tomographic reconstruction demonstrating anterior aortic and mitral–aortic intervalvular fibrosa pseudoaneurysms (*white arrows*). (*B–D*) Orthogonal reconstructions showing adequate movement of the mechanical aortic valve, partial diastolic collapse (*B, D*), and systolic expansion (*C, E*) of the anterior pseudoaneurysm (*yellow arrow heads*), and mitral–aortic intervalvular fibrosa pseudoaneurysm (*blue arrow heads*).

A cardiac and supra-aortic angio-CT revealed two pAs (*Figure 4*): an anterior pA arising from the LVOT, measuring 18 × 5 mm and extending cranially 80 mm (*Figure 4A–C*), and a second non-dissecting periprosthetic pA of the MAIV-F surrounding the 88% of the external perimeter of the prosthesis, with a 21 mm space between the wall and the prosthesis, extending cranially 44 mm (*Figure 4A, C–E*).

In this context, two persistent positive blood cultures for *Streptococcus gallolyticus* met Duke criteria for IE. The patient underwent successful surgical resection of both pAs, with placement of autologous pericardium and replacement of the aortic mechanical valve. Nonetheless, the intervention lasted almost 14 h. Despite preserved ventricular function and normal cardiac output observed on postoperative echocardiographic monitoring, the patient developed hypotension that was unresponsive to high-dose vasopressors. Unfortunately, the patient died 24 h later due to refractory vasoplegic syndrome.

## Patient 3

**Figure 5:**
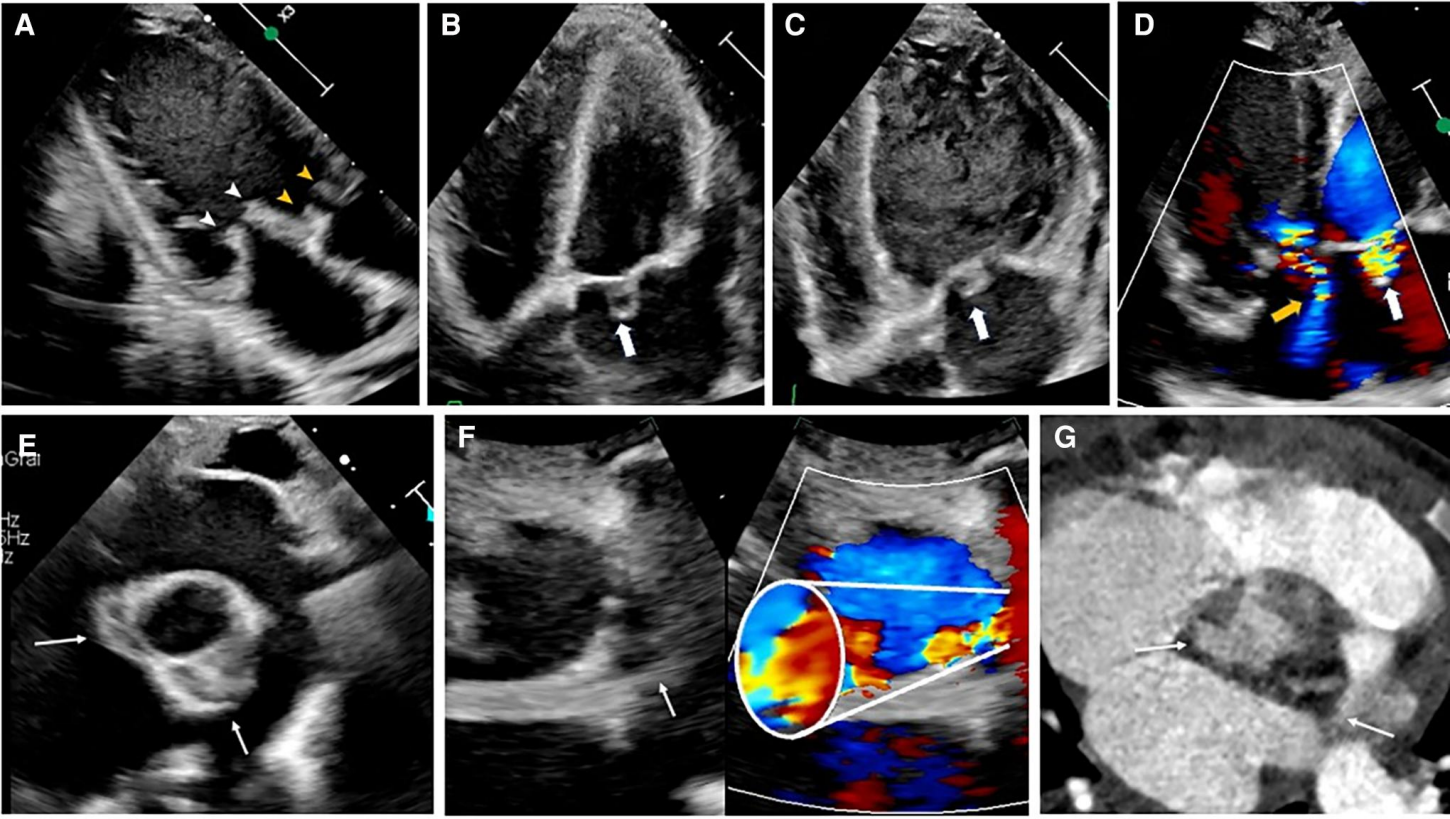
(*A–F*) Transthoracic echocardiography. (*A*) Three-chamber view that shows multiple mobile vegetations in mitral (*white arrow heads*) and aortic (*yellow arrow heads*). (*B–D*) Four-chamber view revealing a mitral pseudoaneurysm (*white thick arrow*) with systolic expansion (*B*), diastolic collapse (*C*), and pulsatile flow (*D*); tricuspid regurgitation is also observed (*yellow arrow in D*). (*E–F*) Paraesternal short axis at the aortic level demonstrating two aortic pseudoaneurysms (*white thin arrows*); zoomed dual image with colour Doppler showing bidirectional flow (*F)*. (*G*) Cardiac angio-tomography depicting both aortic pseudoaneurysms.

**Figure 6:**
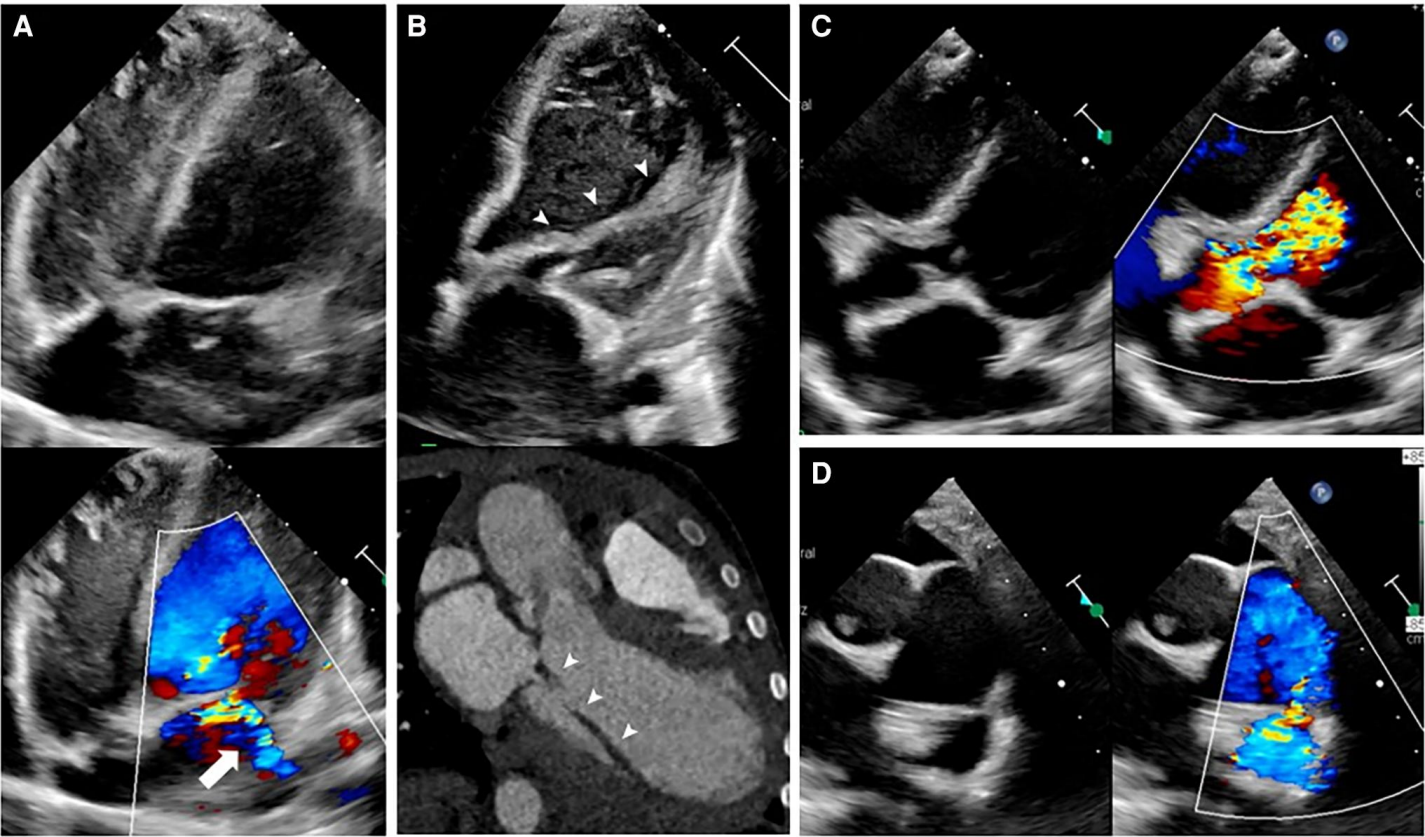
(*A*) Dual four-chamber view showing mitral regurgitation (*white arrow*). (*B*) Echocardiography view (*above*) and tomographic view (*bottom*) demonstrating a hammock mitral valve (*white arrows heads*). (*C*) Dual five-chamber view revealing severe acute regurgitation (*blue arrow*). (*D*) Dual view demonstrating PDA (*yellow arrow*).

A 3-year-old male with a past history of BAV presented with 4 weeks of high-grade fever and hyporexia. On admission, physical examination revealed an aortic murmur. A TTE was performed, which revealed aortic and mitral mobile shaggy masses (*Figure 5A*, Supplementary material online, *Video S5*) and then one pulsatile echo-free space on the atrial face of the anterior mitral leaflet, consistent with a non-perforated mitral pA (*Figure 5B–D*, Supplementary material online, *Video S6*), and two on the aortic root with bidirectional flow (*Figure 6 E–F*). In addition, aortic, tricuspid, and mitral regurgitation were observed (*Figure 6A, C*), the latter secondary to hammock mitral valve (see Supplementary material online, *Video S7*) associated with a patent ductus arteriosus (*Figure 6 B, D*). Later, a computed cardiac angio-CT characterized aortic pAs (*Figure 5G*). Broad-spectrum antibiotic therapy was started and then switched to directed therapy for *Staphylococcus aureus*. The patient developed acute heart failure; haemodynamic support was initiated due to cardiogenic shock. Any surgical intervention was not possible because of prohibitive mortality risk. Unfortunately, 7 days later, the patient died despite maximum medical treatment. *Table 1* shows the main features of the cases.

**Table 1:** Clinical, microbiological, and imaging characteristics of the three cases

Patient	Patient 1	Patient 2	Patient 3
Age	49 years	51 years	3 years
Gender	Female	Male	Male
Symptoms	Fever and dyspnoea	Fever	Fever and hyporexia
Valvular disease	Bicuspid aortic valve and aortic regurgitation	Mechanical aortic valve	Bicuspid aortic valve; hammock mitral valve, aortic and mitral regurgitation
Number and location of vegetations	Two, one in each aortic cusp	None	Four; two in the aortic valve, two in the mitral valve
Microorganism	*Streptococcus infantarius*	*Streptococcus gallolyticus*	*Staphylococcus aureus*
Number and location of pseudoaneurysm	Two; aortic root and mitral–aortic intervalvular fibrosa	Two, aortic root	Two; aortic root and mitral valve
Special features	Different location, and double fistulization of aortic pseudoaneurysm	Dual aetiology suspected (chronic post-surgical and acute infection)	Multivalvular IE; 3 pA; PDA
Surgical management	Reconstruction with autologous pericardium and mechanical valve replacement	Reconstruction with autologous pericardium and mechanical valve replacement	No surgery (prohibitive surgical risk)

## Discussion

The bicuspid aortic valve, aortic regurgitation, and prior Bentall procedure may have contributed to the formation of multiple pAs. The aortic root or MAIV-F are commonly reported sites of infective pAs, with *S. aureus* and streptococci being the most frequent causative organisms,^3^ as observed in our three patients. Only a few case reports of multiple pA were documented,^4,5^ all of which were located in the aortic root. While pAs and the Yin–Ying sign have been described in individual case reports and peripheral vascular contexts, our series emphasizes their simultaneous occurrence at multiple cardiac sites in IE, an uncommon scenario even in tertiary referral centres. Case 1 shows a particularly noteworthy finding: the coexistence of two pAs in distinct anatomical locations—the MAIV-F and aortic root—with double fistulization into both the aortic lumen and LVOT, representing a remarkable and particular presentation.

The leading aetiologies of cardiac pAs are IE and aortic valve surgery, particularly Bentall procedure.^4^ In Case 2, although no post-surgical imaging was available due to loss of the patient during follow-up until the current hospitalization, the pAs likely developed through a dual mechanism: slow postoperative formation followed by accelerated expansion due to superimposed IE. Objectively, however, endocarditis remains the only verified cause in this clinical context, with the prior Bentall procedure being the only risk factor for this complication, highlighting the need for vigilance even years after the procedure.

Multivalvular involvement in IE and the formation of aortic pA^6^ or mitral valve pA^7^ are already uncommon presentations in infants; the coexistence of all three anatomical features in a single paediatric patient (Case 3), in addition to three congenital heart diseases, represents an exceedingly rare and ominous clinical scenario. Initial aortic involvement likely resulted in acute aortic regurgitation, with retrograde volume flow potentially facilitating mechanical and infectious seeding of the mitral valve. This, in turn, led to further valvular destruction and mitral pA formation, ultimately resulting in clinical deterioration and death.

Beyond their rare and unique characteristics of these cases, the diagnostic approach represents another remarkable aspect. Echocardiography plays a crucial role in detecting two important clues during the evaluation of a patient with suspected endocarditis. First, an echo-free space should be identified, characterized by systolic dilatation and diastolic collapse^8^ (a feature also observed on cardiac angio-CT). Second, the presence of flow inside the echo-free space must be demonstrated, either by dynamic aliased Doppler flow (as in the mitral pA of Case 3) or through a distinctive feature known as the Yin–Yang sign.^9^ The name arises from the Chinese symbol of two contrary forces creating balance; in echocardiography, the Yin–Yang sign reflects simultaneous in-and-out or swirling blood flow within the echo-free space, visualized as a bidirectional image on colour Doppler. When these two dynamic clues are present, a pA should be strongly suspected. The detection of flow indicates a communicating channel between the ‘pouch’ and a patent vessel or chamber, which is the main distinction between a pA and a haematoma or abscess.^3^ Another imaging modality to demonstrate the bidirectional flow is through the to-and-fro sign using continuous-wave Doppler; although this was not specifically performed, bidirectional flow within the pAs was clearly demonstrated on colour Doppler, corresponding to the Yin–Yang sign, which remains a key diagnostic feature.

The diagnostic utility of the Ying–Yang sign has been primarily demonstrated in iatrogenic peripheral pAs secondary to percutaneous vascular interventions^10^ and has been briefly mentioned in other cases.^5^ To the best our knowledge, this represents the first case series describing multiple pAs with detailed characterization of such atypical features, thereby highlighting the diagnostic value of this echocardiographic sign in the context of complicated IE and adding important insight into the early recognition of these complex cases. Furthermore, multimodality imaging with 3D-volumetric tomographic reconstructions proved to be an essential tool for accurately assessing pA extension and to guide appropriate surgical interventions.

In conclusion, pA represents a potential life-threatening complication of IE, with the potential for multiple pAs to develop within a single patient. Accurate diagnosis relies on a multimodal imaging approach. Notably, the presence of Yin–Yang sign, paradoxically, should alert clinicians to an underlying structural disruption leading to a loss of balance.

## Lead author biography


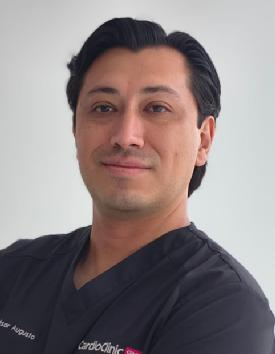


Dr. Rojas graduated in 2022 from the University of Guadalajara with a degree in Internal Medicine. In 2024, he completed a subspecialty in Cardiology at the National Medical Center “November 20” in Mexico City. He currently works as an internist at ISSSTE Tláhuac General Hospital and as a cardiologist, treating patients with cardiovascular diseases at a private clinic. His special interests include heart failure, ischaemic heart disease, cardiovascular imaging, and interventional cardiology.

## Supplementary Material

ytaf589_Supplementary_Data

## Data Availability

The data underlying this article are available in the article and in its online Supplementary material.
